# Functional Training Focused on Motor Development Enhances Gross Motor, Physical Fitness, and Sensory Integration in 5–6-Year-Old Healthy Chinese Children

**DOI:** 10.3389/fped.2022.936799

**Published:** 2022-07-11

**Authors:** Tao Fu, Diruo Zhang, Wei Wang, Hui Geng, Yao Lv, Ruiheng Shen, Te Bu

**Affiliations:** ^1^College of Health and Exercise Science, Tianjin University of Sport, Tianjin, China; ^2^College of Physical Education, Hunan Normal University, Changsha, China

**Keywords:** TGMD-2, preschool, motor skill, physical activity, vestibular function

## Abstract

**Objective:**

Physical inactivity and sensory integration dysfunction are public health concerns among Chinese preschool children. The purpose of this study was to determine the efficacy of a novel functional training program focused on motor development for healthy children aged 5 to 6 years.

**Methods:**

A total of 101 healthy children aged 5 to 6 years in Tianjin were randomly assigned to the experimental group (*N* = 51), which received 12-week functional training featuring essential motor skills, whilst the control group (*N* = 50) continued with their kindergarten-based physical education curriculum. Test of Gross Motor Development-2, national physical fitness measurement, and sensory integration were evaluated before and after the intervention. Children's height, body weight, and the corresponding pre-intervention test scores were utilized as covariates to compare the post-intervention outcomes between the groups.

**Results:**

After the intervention, the experimental group scored considerably higher (*P* < 0.01) on the locomotor composite score, object control composite score, and overall gross motor score than the control group; the experimental group scored higher (*P* < 0.05) on the run, gallop, leap, stationary dribble, kick, striking a stationary ball, overhand throw, and underhand roll motor skill tests than the control group; the experimental group performed considerably better (*P* < 0.01) on the balance beam walking test and sit-and-reach test than the control group; and, the experimental group performed considerably better (*P* < 0.01) on the vestibular function, tactile defensiveness, and proprioception than the control group.

**Conclusion:**

A 12-week functional training focused on motor development effectively enhanced gross motor, physical fitness, and sensory integration in 5–6-year-old healthy Chinese children.

## Introduction

The health of preschool children is critical for healthy growth throughout their lifespan (1). Numerous researches have demonstrated that motor development is closely related to individual health, cognitive ability, emotional wellbeing, and social development, and is a necessary condition for young children to thrive academically (2). Motor development can be classified into a gross motor which involves primarily large muscle groups in the trunk or limbs (e.g., running, jumping), and a fine motor which involves mostly the wrists of the arms or small muscle groups (e.g., drawing, using chopsticks). Gross motor is necessary for the development of motor skills, physical fitness, cognitive, perceptual, and emotional capabilities in early childhood (3, 4). Gross motor is not measured in terms of speed or distance traveled, but rather based on how coordinated and smooth the movement is completed (5). Five to six-years of age in children is a vital developmental stage for preschool children about to join an elementary school, as well as the optimal age for motor development (6). If children's motor skills are fully and evenly developed during this time, it could have a significant impact on their subsequent learning of motor skills in school and even as adults. Otherwise, motor development delay has been shown to be a factor in the developmental delay in children (7).

Functional training originated in rehabilitation and aims to restore the body's fundamental functioning through targeted motor activities on the affected limb. Functional training has now evolved significantly, and its emphasis on the kinetic chain exercise, balance training, and multi-joint functional movements is congruent with fundamental principles of early childhood motor skill development. Generally, researches have proven that functional training (8) or motor development training (9) has a beneficial influence on motor skills in disadvantaged children. However, their combined effect on motor skills in healthy, preschool children is missing in the literature. Given the important role of motor development in early childhood, it is critical to address this research gap.

By the time children reach the age of seven, they should have acquired acceptable levels of competency in fundamental motor skills, as they begin to participate in increasingly specialized physical activities (10). This research integrated functional training and motor development patterns in young children to construct a novel 12-week functional training program focused on motor development for preschool children. The study aimed to establish an experimental basis for promoting motor development, physical activity, and sensory integration in early childhood.

## Methods

### Participants

The research was approved by the Ethics Committee of Tianjin University of Sport and the protocol adhered strictly to the Declaration of Helsinki. Criteria for inclusion include guardians' informed consent and, children were able to collaborate and engage throughout the intervention. Criteria for exclusion include: children without full participation in the intervention, including one missing visit; children who did not participate completely in the pre- and post-intervention assessments of gross motor, physical fitness, and sensory integration; children enrolled in after-school sports or physical fitness classes during the duration of the study; or, children with physical developmental disorders or a history of congenital diseases. A total of 101 eligible children aged 5 to 6 years in Tianjin were randomly assigned to the control and experimental groups. Table 1 summarizes the demographic characteristics of the participants.

**Table 1:** Children's demographic characteristics.

	**Control group**			**Experimental group**		
	**Male (*n* = 26)**	**Female (*n* = 24)**	**Overall (*N* = 50)**	**Male (*n* = 25)**	**Female (*n* = 26)**	**Overall (*N* = 51)**
Height (cm)	115.7 ± 4.2	114.4 ± 4.3	115.1 ± 4.2	113.6 ± 3.0	114.9 ± 4.4	114.2 ± 3.8
Body weight (kg)	20.6 ± 4.0	20.0 ± 2.6	20.3 ± 3.4	19.2 ± 2.2	20.1 ± 2.9	19.7 ± 2.6

*Data are expressed as mean ± standard deviation*.

### Experimental Design

This research was conducted between October and December of 2021. In October and November, the control and experimental groups were primarily engaged in outdoor activities. Due to the cold winter weather in Tianjin, both groups were primarily engaged in indoor activities in December. Outcome tests were administered 1 week before to the first intervention session and 1 day following the last session.

The control group followed a kindergarten-based physical education curriculum, with each lesson lasting 40–50 min and focusing on group games, gymnastics, and free play as the primary modes of movement. Table 2 summarizes the three primary components of the kindergarten self-designed curriculum, which include rhythmic exercise, games, and free activities for children. In brief, the curriculum covers a limited number of motor skills, the movements are simple, and there is no discernible division of difficulty levels. This curriculum is designed to promote children's interest in physical education and fulfill the Ministry of Education's requirement for physical activity. The control group received the lesson once a week during weeks 1–8 and twice a week during weeks 9–12.

**Table 2:** Description of the kindergarten-based physical education curriculum.

**Curriculum contents**	**Duration**	**Activities**
Warm-up	3–5 min	Jogging, rotational movement
Rhythmic exercise	5–10 min	Standing rhythmic exercise, or stationary drill
Games	7–10 min	Slapping ball, relay race, single leg jump, cart pushing run
Free play	10–15 min	Unstructured free activities, playground slides
Relaxation	3–5 min	Body shaking exercise
Stretching	1–3 min	Low-impact upper and lower body stretching

The experimental group participated in a functional training program meant to promote motor development. In constructing the functional training program, existing guidelines (11) and published protocol (5) were reviewed, as well as the developmental characteristics (12), cognitive qualities (13), Chinese-specific movement patterns of 5–6-year-old children (14). The protocol and components of the functional training program were refined by consulting with experts and teachers in preschool education. The intervention period was replaced by the same time as the kindergarten-based physical education curriculum, and each training session lasted 40–50 min. Table 3 summarizes the main activities in the functional training program. The program is centered on motor skills and is tailored to the child's cognitive and emotional needs, with mini-games such as tennis, football (soccer), and basketball used to interest young children in training. Each training session begins with a preparation phase, including warm-up, neuromuscular activation, dynamic stretching, and core muscle activation. The main training phase has consisted of two modules. This first module is devoted to motor skill development, with an emphasis on walking, jumping, crawling, throwing, pushing, and catching. The second module focuses on physical fitness, with an emphasis on balance, agility, and endurance. Each training session concludes with a phase of motor skill consolidation and static stretching. The experimental group received a 1–4 week period for basic skill acquisition with one training per week, a 5–8 week period for basic skill consolidation with one training per week, and a 9–12 week period for skill strengthening with two training per week.

**Table 3:** Description of the functional training program.

**Exercise modules**	**Duration**	**Themes**	**Activities**
**Warm-up phase**			
Warm-up	1–3 min	Train on the move	Children stand in a line while moving forward together
		Plane takeoff	Lateral raise of both arms and jog forward
Neuromuscular activation	1–3 min	High knees	Lift one knee to the chest and then the other
Dynamic stretching	1–3 min	Stretch in motion	Walk lunge step, bring leg up high to chest on each step
Core muscle activation	1–3 min	Tunnel pass	Crawl through a play tunnel
		Flutter kicks	Alternately raise and lower legs from floor
**Main training phase**			
Motor skill development	10–15 min	Walk	Obstacle curved walk, reactive direction, squat walk etc.
Physical fitness	7–10 min	Run	Chasing and fleeing run, reactive speed, join hands run etc.
		Jump	Multi-directional jump, jumping rope, straddle jump, etc.
		Mini-ball games	Kick, throw, catch, slap, dribble, tap, small-sided games
		Coordination	Skip with a hula hoop, over and under relay, group hug etc.
**Cool-down phase**			
Motor skill consolidation	1–3 min	Whac-A-Mole	Children are told what color the barrel is, and they touch it
		Pass the parcel	Pass around a ball while music is playing
Static stretching	1–3 min	Animal imitation	Practice various static postures

During the intervention period, both groups received similar types and frequencies of general education in kindergarten, including music and arts curriculum.

### Instrument

Gross motor was assessed using the Chinese children's validated Test of Gross Motor Development-2 (TGMD-2) (15). TGMD-2 is composed of locomotor and object control domains. Each domain contains six skill tests, for a total of 12 fundamental movement skills. Each skill comprises 3–5 scoring criteria: performed the standard or not performed the standard, which are assigned one or zero points. Two domains' scores are added together to provide a composite score for each domain. After that, the scores for two domains can be added together to obtain an overall gross motor score.

Between 9:00 and 10:30 a.m., the same group of testers administered the physical fitness test in the kindergarten gym and playground, according to the test criteria outlined in the National Physical Fitness Measurement Standards Manual (Preschool Children Version) (16). The test battery gauges different components of fitness, including the standing long jump test, balance beam walking test, tennis throwing test, sit-and-reach test, 10-m shuttle run test, and double-leg timed hop test. A detailed measurement procedure can be found in an independent evaluation of the test (17).

Sensory integration was evaluated using the Child Sensory Integration Scale (18), which was established based on American psychiatrist Jean Ayres's classic sensory integration theory and has been validated in Chinese children aged 3 through 11 years (19). The sensory integration test requires parental cooperation, and due to the very subjective nature of parental self-assessment, the same rehabilitation therapist must help parents in completing an online questionnaire before and after the intervention to guarantee the scale is objectively consistent. The scale consists of the following five domains: vestibular function (14 items), which evaluates gross motor; tactile defensiveness (21 items), which evaluates emotional stability and a tendency to over-defend; proprioception (12 items), which evaluates body's proprioception and balance coordination; learning ability (8 items), which evaluates learning deficits due to poor sensory integration; and, issues particular to children beyond the age of 10 (8 items), which was not assessed in the present study population. Each of the 50 items was scored using a five-point Likert scale as follows: 5 = never (the child never responds in this manner when presented with the opportunity); 4 = seldom (the child responds occasionally in this manner); 3 = occasionally (the child responds sometimes); 2 = frequently; 1 = always (the child responds in the manner noted whenever presented with the opportunity). Total scores for each domain were theoretically between 8 and 105, with higher scores indicating better performance.

### Statistics

The IBM SPSS Statistics 24.0 was used for the statistical analysis. A Shapiro-Wilk test was used to confirm data normality. First, a *t*-test was used to evaluate whether there were pre-intervention test differences between the groups. Second, a *t*-test was used to evaluate the training effect within the groups. Third, an analysis of covariance was used to evaluate the training effect between the groups, using the height (except for sensory integration), body weight (except for sensory integration), and corresponding pre-intervention test scores as continuous covariates. Results for the sit-and-reach test were compared using the paired Wilcoxon test within groups, and the Mann-Whitney *U* test between groups. *P* < 0.05 was considered statistically significant.

## Results

Table 4 summarizes the gross motor test results. Before the intervention, the control group scored considerably higher (*P* < 0.01) on the kick test than the experimental group. There were no significant differences (*P* > 0.05) in any of the other gross motor tests between the groups before the intervention.

**Table 4:** Results of gross motor.

**Domain**	**Test battery**	**Control group (*****N*** **=** **50)**		**Experimental group (*****N*** **=** **51)**	
		**Pre-intervention**	**Post-intervention**	**Pre-intervention**	**Post-intervention**
Locomotor	Run	6.86 ± 1.01	6.74 ± 1.07	6.76 ± 0.99	7.16 ± 0.97^‡^^¶^
	Horizontal jump	6.72 ± 1.21	6.74 ± 1.01	6.49 ± 0.76	7.06 ± 0.79^§^
	Hop	8.30 ± 1.06	8.56 ± 0.99	8.18 ± 0.74	8.75 ± 0.87^§^
	Gallop	3.58 ± 0.91	3.96 ± 0.88^‡^	3.71 ± 0.83	5.29 ± 0.97^§^^||^
	Leap	6.16 ± 0.77	6.44 ± 0.97	6.06 ± 0.88	6.96 ± 0.87^§^^||^
	Slide	7.16 ± 0.84	7.42 ± 0.95	7.08 ± 0.87	7.57 ± 1.29^‡^
Locomotor composite score		38.78 ± 2.31	39.86 ± 2.39^‡^	38.27 ± 2.69	42.78 ± 2.11^§^^||^
Object control	Stationary dribble	5.20 ± 1.11	5.62 ± 0.89^‡^	5.04 ± 0.98	6.14 ± 0.75^§^^¶^
	Kick	7.00 ± 0.78	6.08 ± 0.83^§^	6.51 ± 0.88^†^	6.86 ± 0.72^‡^^||^
	Catch	4.98 ± 0.65	5.00 ± 0.81	4.69 ± 0.86	5.31 ± 0.88^§^
	Striking a stationary ball	5.04 ± 1.41	5.12 ± 0.82	5.41 ± 0.90	6.98 ± 1.03^§^^||^
	Overhand throw	5.44 ± 0.97	5.02 ± 0.71^‡^	5.20 ± 1.02	6.22 ± 0.92^§^^||^
	Underhand roll	5.40 ± 0.95	5.12 ± 0.82	5.35 ± 0.96	6.22 ± 0.76^§^^||^
Object control composite score		33.06 ± 2.67	31.96 ± 1.96^‡^	32.20 ± 2.09	37.73 ± 2.32^§^^||^
Overall gross motor score		71.84 ± 3.33	71.82 ± 3.59	70.47 ± 3.09*	80.51 ± 3.35^§^^||^

Data are expressed as mean ± standard deviation. Before the intervention, control group vs. experimental group: ^*^ P < 0.05,

† *P < 0.01; within the control/experimental group, pre-intervention vs. post-intervention*:

‡ *P < 0.05*,

§ *P < 0.01; after the intervention, control group vs. experimental group*:

¶ *P < 0.05*,

|| *P < 0.01*.

Within the control group, the post-intervention locomotor composite score was higher (*P* < 0.05), with a higher (*P* < 0.05) gallop test score and no significant changes (*P* > 0.05) in the scores of the other five locomotor tests; the post-intervention object control composite score was lower (*P* < 0.05), with a lower (*P* < 0.05) striking a stationary ball test score, lower (*P* < 0.05) overhand throw test score, and considerably lower (*P* < 0.01) kick test score; and, there was no change (*P* > 0.05) in the overall gross motor score.

Within the experimental group, the post-intervention locomotor composite score, object control composite score, and overall gross motor score were all considerably higher (*P* < 0.01); and, all post-intervention locomotor and object control test scores were higher (*P* < 0.05).

After the intervention, the experimental group scored considerably higher (*P* < 0.01) on the locomotor composite score, object control composite score, and overall gross motor score than the control group; the experimental group scored higher (*P* < 0.05) on the run and stationary dribble tests than the control group; and, the experimental group scored considerably higher (*P* < 0.01) on the gallop, leap, kick, striking a stationary ball, overhand throw, and underhand roll tests than the control group.

Table 5 summarizes the physical fitness test results. Before the intervention, there were no significant differences (*P* > 0.05) in any of the physical fitness tests between the groups.

**Table 5:** Results of physical fitness.

**Test battery**	**Control group (*****N*** **=** **50)**		**Experimental group (*****N*** **=** **51)**	
	**Pre-intervention**	**Post-intervention**	**Pre-intervention**	**Post-intervention**
Standing long jump test (cm)	100.84 ± 14.27	101.64 ± 11.30	99.78 ± 8.24	103.43 ± 12.01^‡^
Balance beam walking test (s)	9.61 ± 1.55	9.68 ± 1.10	10.00 ± 1.74	8.98 ± 0.87^§^^||^
Tennis throwing test (m)	4.36 ± 1.56	4.76 ± 1.76	4.05 ± 1.24	4.85 ± 1.40^§^
Sit-and-reach test (cm)	3.36 ± 5.64	4.32 ± 6.29	4.84 ± 6.04	7.57 ± 5.49^‡^^||^
10-m shuttle run test (s)	8.54 ± 1.10	7.91 ± 1.06^§^	8.63 ± 0.93	7.89 ± 0.68^§^
Double-leg timed hop test (s)	6.67 ± 1.65	6.12 ± 1.07	7.11 ± 1.40	5.80 ± 0.66^§^

*Data are expressed as mean ± standard deviation. Within the control/experimental group, pre-intervention vs. post-intervention*:

‡ *P <0.05*,

§ *P <0.01; after the intervention, control group vs. experimental group*:

|| *P <0.01*.

Within the control group, the post-intervention 10-m shuttle run test was considerably better (*P* < 0.01). Within the experimental group, the post-intervention standing long jump test and sit-and-reach test were better (*P* < 0.05), and the post-intervention balance beam walking test, tennis throwing test, 10-m shuttle run test, and double-leg timed hop test were considerably better (*P* < 0.01).

After the intervention, the experimental group performed considerably better (*P* < 0.01) on the balance beam walking test and sit-and-reach test than the control group.

Table 6 summarizes the sensory integration test results. Before the intervention, there were no significant differences (*P* > 0.05) in any of the sensory integration domains between the groups.

**Table 6:** Results of sensory integration.

**Domain**	**Control group (*****N*** **=** **50)**		**Experimental group (*****N*** **=** **51)**	
	**Pre-intervention**	**Post-intervention**	**Pre-intervention**	**Post-intervention**
Vestibular function	57.26 ± 8.05	58.06 ± 7.19	59.00 ± 5.95	62.92 ± 3.37^§^^||^
Tactile defensiveness	94.60 ± 9.44	94.76 ± 9.66	94.63 ± 10.03	99.40 ± 5.69^§^^||^
Proprioception	55.10 ± 5.32	54.80 ± 5.19	56.20 ± 4.00	57.35 ± 3.46^||^
Learning ability	36.52 ± 4.67	36.40 ± 4.31	34.80 ± 6.12	37.73 ± 2.61^§^

*Data are expressed as mean ± standard deviation. Within the control/experimental group, pre-intervention vs. post-intervention*:

§ *P < 0.01; after the intervention, control group vs. experimental group*:

|| *P < 0.01*.

Within the control group, there was no change (*P* > 0.05) in the sensory integration. Within the experimental group, the post-intervention vestibular function, tactile defensiveness, and learning ability were considerably improved (*P* < 0.01).

After the intervention, the experimental group performed considerably better (*P* < 0.01) on the vestibular function, tactile defensiveness, and proprioception than the control group.

## Discussion

Motor development is the process through which individuals go from unstructured, untrained movements to regular, complicated, and deliberate movements (20). At the age of five, the fundamental motor skills gradually improve, and various motor skills gradually develop at the age of six (21). Given that gross motor activities are fundamental motor skills that are developed during the early stages of children's growth, the development of gross motor skills can be prioritized. In this study, kindergarten-based physical education curriculum can only improve certain crucial gross motor and physical fitness characteristics, while having minimal effect on sensory integration. Following a 12-week functional training program focused on motor development, healthy children aged 5 to 6 years performed better in terms of gross motor, physical fitness, and sensory integration. The implication is clear that kindergartens and communities should consider physical education programs with scientific rigor, such as this functional training program, in order for preschool children to develop important motor skills in a timely and sufficient manner, which could have significant effects on their physical and cognitive development.

The kindergarten-based physical education curriculum improved children's locomotor skills but not their object control skills, and the effect on children's gross motor development is not significant. The control group performed significantly better on the pre-intervention kick test than the experimental group. The reason for this could be that the kindergarten-based physical education curriculum had already begun teaching some children football classes. Consequently, some children in both the control and experimental groups were exposed to the kicking motion, resulting in disparities in their gross motor test scores before the intervention. In terms of the higher locomotor composite score, the regular parent-child sporting games held in the kindergarten establish a hurdle race, which is similar to the gallop test in the TGMD-2. As a result, the kindergarten-based physical education curriculum repeatedly consolidates the gallop movement skill while preparing for the sporting games and practicing hurdles, which has a beneficial effect on children's locomotor skills.

The functional training program integrates fundamental movement and balance skills and motor coordination activities for gross motor development. After the 12-week intervention, the locomotor composite score, object control composite score, and overall gross motor score were all higher in the experimental group, demonstrating that the functional training program focused on motor development can help preschool children's gross motor development. Meanwhile, the functional training program incorporates group-based ball games into the workout. Ball activities are very interactive and can not only pique young children's interest, but also improve their observation, judgment, and agility (22). Additionally, Wang et al. (14) has revealed that the rate at which children develop motor skills is related to the frequency with which they play with peers in Chinese preschool children aged 3 to 6 years. Epidemiological researches have generally found a suboptimal level of physical activity in Chinese preschool children aged 3 to 6 years (23, 24). Given the association between physical activity and motor skills, it is not unexpected that Chinese preschool children without adequate physical activity have suboptimal motor development. After adjusting for covariates, Rao et al. (25) discovered that the motor skill development scores of 4-year-old children were significantly lower in 2017 than in 2013. Our findings should be insightful for researchers and practitioners when designing motor development programs for preschool children in kindergartens or communities.

Early childhood is a vital period for physical fitness development. Adequate exercise can aid in the normal development of children's various organs, and physical fitness is a critical indicator of children's health (26). If young children lack physical activity and health-related fitness, they may be more susceptible to chronic diseases such as type 2 diabetes (27) and hypertension (28). Thus, regular physical activity is critical to developing motor skills in early childhood and promoting health throughout their life cycle (29). Meanwhile, motor skill development is a significant predictor of greater levels of health-related physical fitness and physical activity behaviors in preschool children, as well as improved health outcomes (30). It has been demonstrated that the development of 3–6-year-old children's motor skills is positively correlated with their physical health (31). Longitudinal studies also have demonstrated that children's ability to acquire motor skills continues to influence their development of physical fitness and health (32). This study provided evidence that the kindergarten-based physical education curriculum can only help children improve their speed quality, implying that the curriculum cannot address all facets of children's physical fitness. This paucity of high-quality physical activity in Chinese kindergartens is consistent with previous research. For example, Hu et al. (12) evaluated the quality of outdoor play in 91 Chinese kindergartens. They reported insufficient opportunities for outdoor play and a lack of physical activity among children aged 3 to 6 years. In comparison, the functional training program focused on motor development increased all physical characteristics of preschool children, with more noticeable outcomes in balance and flexibility. The functional training program is composed of supervised motor skill activities that can help children improve their sensitivity to external stimuli, develop new conditioned reflexes, and thus foster the development of their body's flexibility and coordination. Recent epidemiological studies on the incidence of developmental coordination disorder in Chinese children found that the overall incidence was 3.4%, and suspected cases were 5.4%, among 3–5-year-old children (33); and the overall incidence was 5.5%, and suspected cases were 10.4%, among 3–10-year-old children (34). This functional training program is particularly relevant to contemporary Chinese society for the healthy development of early childhood.

Sensory integration is critical for children's capacity to learn and social development across their lifespan (35). Children with sensory integration dysfunction are more vulnerable to external influences during movement, exhibiting intense mood swings, decreased self-control, a difficulty to maintain bodily balance, and, in severe cases, aggression. It is a significant public health problem worldwide, with a prevalence of sensory integration dysfunction ranging from 36.94% (36) to 55.8% (37) in Chinese preschool children aged 3 to 6 years. In this study, the functional training program focused on motor development was more effective at improving children's sensory integration than kindergarten-based physical education curriculum. This outcome is consistent with the literature. In Chinese children aged 3 to 6 years, it has been validated that TGMD-2 had a highly significant positive correlation with children's static balance, dynamic balance, and proprioception (38). On the one hand, the greater the risk of sensory integration dysfunction, the more difficult it is to acquire motor skills (39); on the other hand, the locomotor and object control of gross motor skills are correlated with vestibular function and proprioception in sensory integration (40). Functional training is structured around better neuromuscular responses, such as 2-foot hop, which aids children's spatial orientation, improve their balance, and stimulate their vestibular and proprioceptive senses. The present results suggest that a 12-week functional training can improve children's general sensory development, and that establishing a motor development-focused program could be a successful early life educational intervention strategy.

Notably, the functional training program enhanced the learning ability within the experimental group. Motor development is favorably associated with young children's learning ability. A regression analysis of 4–5-year-old children in the Northeast of England discovered that motor skill acquisition improved the number of children who are prepared to enter elementary school and that promoting gross and fine motor skills may increase the number of children who meet entry requirements and are more likely to achieve better educational outcomes (41). This observation was similarly validated among Chinese preschool children. Recently, Chou et al. (42) showed that 4–5-year-old children enrolling in kindergartens with better physical fitness programs had better executive function, which was associated with better academic skills. Furthermore, children with better motor skills had greater executive functions and obtained more academic skills in kindergartens (42). Thus, not only does the functional training program help preschool children's physical fitness, but it may also have a beneficial effect on their long-term learning ability.

This study has two limitations. First, there are six recommended self-reported levels of exercise intensity for Chinese preschool children aged 5 to 6 years, corresponding to an average heart rate of <120, 120, 120–140, 140, 140–160, and >160 beats**·**min^−1^ (43). Tan et al. (44) suggested that the target heart rate for physical activity in Chinese children aged 3 to 6 years should range between 126.3 and 165.8 beats**·**min^−1^. As a result, this study designed the functional training program maintaining a target heart rate of 120–160 beats**·**min^−1^. During the pilot test, both the kindergarten-based physical education curriculum and the functional training program fell within this target heart rate, and there were no statistically significant differences between the two exercise intensities. Due to the impossibility of concurrently recording all heart rate responses in the research context, we cannot rule out the possibility that the exercise intensity throughout the intervention period was different at certain periods. Second, this study examined the effect of a functional training program on 5–6-year-old healthy children, restricting the capacity to generalize these findings to other age groups or children with physical developmental disorders.

In conclusion, a 12-week functional training program focused on motor development improved gross motor, physical fitness, and sensory integrity in healthy Chinese preschool children. This study provided evidence that functional training can be used to accelerate motor development in this age group and has resulted in considerable advancements in a healthy Chinese population. Based on these findings, we recommend expanding this novel functional training program to kindergartens, schools, and communities in order to promote the healthy development of preschool children.

## Data Availability Statement

The raw data supporting the conclusions of this article will be made available by the authors, without undue reservation.

## Ethics Statement

The studies involving human participants were reviewed and approved by Tianjin University of Sport. Written informed consent to participate in this study was provided by the participants' legal guardian/next of kin.

## Author Contributions

TF and DZ: conceptualization, methodology, and writing—original draft preparation. DZ, WW, HG, YL, and RS: data collection. TF, DZ, WW, HG, YL, RS, and TB: writing—review and editing. TF: project administration and funding acquisition. All authors have read and agreed to the published version of the manuscript.

## Funding

This research was funded by the Humanities and Social Science Fund of the Ministry of Education of China an empirical study on the value, characteristics and curriculum system development of early childhood sports, grant number 20YJA890004.

## Conflict of Interest

The authors declare that the research was conducted in the absence of any commercial or financial relationships that could be construed as a potential conflict of interest.

## Publisher's Note

All claims expressed in this article are solely those of the authors and do not necessarily represent those of their affiliated organizations, or those of the publisher, the editors and the reviewers. Any product that may be evaluated in this article, or claim that may be made by its manufacturer, is not guaranteed or endorsed by the publisher.
